# Inferring miRNA sponge modules across major neuropsychiatric disorders

**DOI:** 10.3389/fnmol.2022.1009662

**Published:** 2022-10-28

**Authors:** Rami Balasubramanian, P. K. Vinod

**Affiliations:** Centre for Computational Natural Sciences and Bioinformatics, International Institute of Information Technology, Hyderabad, India

**Keywords:** non-coding RNAs, network biology, co-expression network, miRNA sponge modules, neuropsychiatric disorders (NPD)

## Abstract

The role of non-coding RNAs in neuropsychiatric disorders (NPDs) is an emerging field of study. The long non-coding RNAs (lncRNAs) are shown to sponge the microRNAs (miRNAs) from interacting with their target mRNAs. Investigating the sponge activity of lncRNAs in NPDs will provide further insights into biological mechanisms and help identify disease biomarkers. In this study, a large-scale inference of the lncRNA-related miRNA sponge network of pan-neuropsychiatric disorders, including autism spectrum disorder (ASD), schizophrenia (SCZ), and bipolar disorder (BD), was carried out using brain transcriptomic (RNA-Seq) data. The candidate miRNA sponge modules were identified based on the co-expression pattern of non-coding RNAs, sharing of miRNA binding sites, and sensitivity canonical correlation. miRNA sponge modules are associated with chemical synaptic transmission, nervous system development, metabolism, immune system response, ribosomes, and pathways in cancer. The identified modules showed similar and distinct gene expression patterns depending on the neuropsychiatric condition. The preservation of miRNA sponge modules was shown in the independent brain and blood-transcriptomic datasets of NPDs. We also identified miRNA sponging lncRNAs that may be potential diagnostic biomarkers for NPDs. Our study provides a comprehensive resource on miRNA sponging in NPDs.

## Introduction

Neuropsychiatric disorders (NPDs) are considered the leading cause of disease burden worldwide. The global number of disability-adjusted life years (DALYs) due to psychiatric disorders increased from 80.8 million to 125.3 million (Ferrari, 2022). Psychiatric disorders affect cortical functions, including mood, behavior, perception, and cognition (Sullivan and Geschwind, 2019). Autism spectrum disorder (ASD), schizophrenia (SCZ), and bipolar disorder (BD) are the common NPDs leading to long-term disabilities, and comprehending the pathogenesis of these disorders is critical. Genetic variants of NPDs are well known. However, little is known about the neurobiological mechanisms by which genetic variants interact with environmental and epigenetic risk factors in the brain. The brain transcriptome, a quantitative phenotype, provides disease-related signatures and associated molecular pathways across the NPD. In ASD, SCZ, and BD, patterns of shared and distinct gene expression perturbations are observed. Glial cell differentiation and fatty acid metabolism pathways are upregulated across the three conditions (Gandal et al., 2018a).

The largest class of transcripts in the human genome, non-coding RNAs(ncRNAs), are linked to various complex phenotypes. However, only a few ncRNAs have been functionally characterized in NPDs. ncRNAs show heterogeneity across the human cell types and tissue. Tissue-specific transcriptome aids in studying transcriptional regulation and non-coding genome function. Understanding the dysregulation of non-coding RNAs in the brain during disease conditions can help in the development of more effective diagnostic and treatment strategies (Gandal et al., 2018a). The competing endogenous RNA (ceRNA) hypothesis suggests that long non-coding RNAs(lncRNAs) can act as microRNA sponges by competitively binding to microRNAs (miRNA) *via* microRNA response elements (MREs) and indirectly controlling the expression level of mRNAs. The crosstalk between miRNAs, lncRNAs, and mRNAs forms a miRNA sponge or ceRNA network (Salmena et al., 2011). The differential expressed miRNAs, lncRNAs and mRNAs are commonly used to construct disease ceRNA networks based on putative or predicted lncRNA-mRNA and miRNA-mRNA interactions. Some of the other existing methods relate miRNA expression and their co-regulated genes by considering the pair-wise correlation between two ceRNAs (positive) and the ceRNAs and their miRNA (negative) (Zhang et al., 2022). Partial association-based methods are also used to model the relationship between ceRNAs and their miRNA expressions directly. List et al. (2019) developed a sparse partial correlation-based sponge network inference method that uses gene expression and miRNA target binding sites.

Generally, the ceRNA networks are large, so identifying the subnetwork or modules may help to understand the functional role of ceRNAs and identify important genes by the guilt-by-association principle. Different methods have been proposed for identifying miRNA sponge modules, including network-based clustering and matrix factorization. Zhang J. et al. (2020) proposed a modular approach, lncRNA-related miRNA sponge modules (LMSM), based on the hypothesis that co-expressed lncRNAs can compete with a group of mRNAs for binding with miRNA. Zhang et al. (2022) assessed the performance of different methods to obtain biologically meaningful miRNA sponge modules based on the efficiency of methods in capturing the disease molecular signatures. In their comparison study, the LMSM approach identified disease-associated diagnostic and prognostic modules in a higher percentage than other methods.

Several studies have explored the novel role of the ceRNA network in different cancers. A comprehensive resource of interactions of miRNA sponging for cancers is available, including Pan-ceRNADB, SPONGEDB, and ENCORI starbase databases (Li et al., 2014; Xu et al., 2015; Hoffmann et al., 2021). On the other hand, only a few studies investigate the miRNA sponging in neurodegenerative and NPDs. These studies are restricted to inferring ceRNA primarily based on differential expressed lncRNAs and mRNA (Zhou et al., 2019; Zhang X. et al., 2020, Zhang J. et al., 2021; Zhang Y. et al., 2021; He et al., 2021; Sabaie et al., 2021a, b). There is a need to infer miRNA sponge modules by considering the expression of miRNA, lncRNAs, and mRNA.

In this study, we predicted the miRNA sponge modules of pan-neuropsychiatric disorders, including ASD, SCZ, and BD, using a modular approach. A co-expression network was constructed using RNA-Seq data of pan-neuropsychiatric disorders. The candidate miRNA sponge modules were identified based on sharing of miRNA binding sites and the sensitivity canonical correlation (Zhang J. et al., 2020). We also investigated the biological processes and molecular pathways associated with miRNA sponge modules and identified potential lncRNAs that can serve as diagnostic markers to distinguish NPDs. Our study provides a comprehensive resource on miRNA sponging in NPDs.

## Materials and Methods

### Data processing

**Figure 1:**
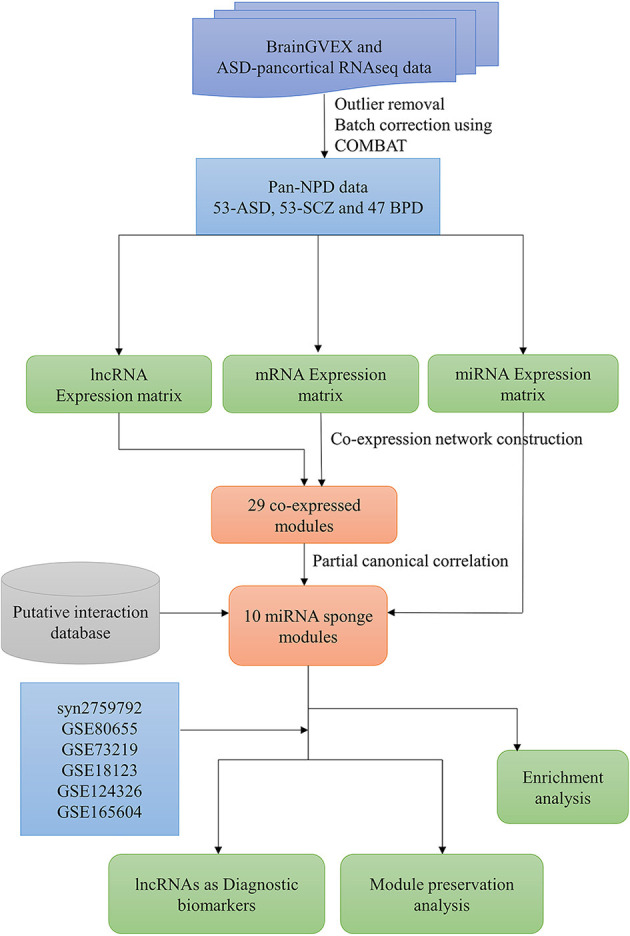
The workflow to infer the lncRNA-related miRNA sponge modules in pan-neuropsychiatric orders.

The overall pipeline of the work is shown in Figure 1. The pre-processed RNA-Seq datasets from the BrainGVEX study and ASD-pan cortical study were retrieved from Synapse with accession numbers syn4590909 and syn11242290, respectively (Gandal et al., 2018b). BrainGVEX contains the prefrontal cortex of post-mortem samples (53 Schizophrenia and 47 bipolar disorder patients). ASD pan-cortical data includes 53 samples from Brodmann Area 4/6, Brodmann Area 38, Brodmann Area 7, and Brodmann Area 17 (Supplementary Table S1). We also retrieved common mind consortium data (syn2759792) and GEO datasets of post-mortem brains of ASD, SCZ, and BD patients for validation (Irimia et al., 2014; Ramaker et al., 2017; Gandal et al., 2018b; Supplementary Table S2). Further, blood-based samples of ASD, SCZ, and BD patients were retrieved for the comparative study (Kong et al., 2012; Krebs et al., 2020; Gatta et al., 2021). For RNA-Seq data, read counts were normalized using conditional quantile normalization (cqn R package) (Hansen et al., 2012). Genes that are not expressed in more than 50% of samples were removed. The microarray data were processed using Robust MultiChip Average (RMA) algorithm in the R (affy package) (Irizarry et al., 2003). This package does background correction, quantile normalization, and probe summarization (Gautier et al., 2004). The outliers were removed based on a standardized network connectivity score (Z score < −2) (Oldham et al., 2012). A pan-neuropsychiatric disorder (pan-NPD) expression data was created by combining BrainGVEX and ASD pan-cortical datasets. The Combat function (sva package v3.26.0) in R was used to correct batch effects (Johnson et al., 2007).

### Weighted gene co-expression network analysis (WGCNA)

The gene co-expression network of pan neuropsychiatric disorder was constructed using the WGCNA package in R to organize the transcriptome into functional modules (Langfelder et al., 2008). At first, a correlation matrix (S_ij_) was constructed based on the Pearson correlation between gene expression and its sign retained by a linear transformation. A weighted adjacency matrix was constructed with the function ${\text{a}}_{\text{ij}}={\text{S}}_{\text{ij}}^{\beta }$. A scale-free topology criterion was used for choosing the soft threshold power β. The square of the correlation *R*^2^ was used to measure the goodness of scale-free topology. Saturation is reached where the *R*^2^ value is greater than 0.8 in the power-law curve. The power β = 18 was considered for the analysis, and the resulting adjacency matrix was transformed into a topological overlap matrix (TOM), and a dendrogram was constructed using 1-TOM as a distance measure (Zhang and Horvath, 2005). The genes were clustered into modules using a dynamic tree-cut algorithm with a minimum module size of 50. The module eigengene (ME) expression values representing the maximum variation of module genes were obtained using singular value decomposition. Co-expressed modules with at least three lncRNAs were considered for further analysis.

### Canonical correlation-based approach for miRNA sponge module identification

Zhang J. et al. (2020) proposed a modular approach to identify lncRNA-related miRNA sponge modules by integrating gene expression data and miRNA-target interactions. The putative miRNA-mRNA targets were retrieved from miRTarBase, TarBase, TargetScan, and miRCode (Sethupathy et al., 2006; Jeggari et al., 2012; McGeary et al., 2019; Huang et al., 2020). The putative miRNA-lncRNA targets were retrieved from DIANA-LncBase, NpInter, and miRCode (Jeggari et al., 2012; Karagkouni et al., 2020; Teng et al., 2020). A miRNA-target matrix was created from these databases.

A co-expression module is considered a miRNA sponge module if the group of lncRNAs and mRNAs in the co-expression module: (1) have significant sharing of miRNAs; (2) have a high canonical correlation between their expression levels; and (3) have significant sensitivity canonical correlation. We performed the hypergeometric test to identify the modules with significant sharing of miRNAs between the group of lncRNAs and mRNAs in the module based on miRNA-target interactions. The significance of the hypergeometric test is calculated as


(2) $$p-value=1-\frac{\sum_{i_{1=0}}^{L_{1^{-1}}}\left(\begin{array}{c} M_{1}\\ i_{1} \end{array}\right)\left(\begin{array}{c} N_{1}-M_{1}\\ K_{1}-i_{1} \end{array}\right)}{\left(\begin{array}{c} N_{1}\\ K_{1} \end{array}\right)}$$

In Equation 1, N_1_ is the number of miRNAs in the expression data, and M_1_ and K_1_ are the total number of miRNAs interacting with the group of lncRNAs and mRNAs in the module, respectively. L_1_ is the number of miRNAs shared by the lncRNAs and mRNAs in the co-expression module. Modules with a *p*-value < 0.05 were filtered as modules with significant sharing of miRNAs. For the filtered modules, the canonical correlation (CClncR-mR) of lncRNAs and the mRNAs in the module was calculated using Equation 2.


(3) $$CC_{l}{}_{ncR-mR}=\frac{a^{T}\sum XY^{b}}{\sqrt{a^{T}\sum XY^{a}}\sqrt{b^{T}\sum XY^{b}}}$$

The vectors $X={\left(x_{1},x_{2},\ldots ,x_{3}\right)}^{T}$ and $Y={\left(y_{1},y_{2},\ldots ,y_{3}\right)}^{T}$ in Equation 2 represent the group of lncRNAs and the group of mRNAs in a module, respectively. ∑XX, ∑YY and ∑XY are the variances and cross-variance matrices calculated from the expression of X and Y. a (a ∈ ℝ*^m^*) and b (b ∈ ℝ*^n^*) are the canonical vectors to maximize the correlation.

The miRNAs shared by the lncRNAs and mRNAs in a module strongly influence the competition between RNAs. Sensitivity canonical correlation (SCC) explains the influence of miRNA expression over the lncRNAs and mRNAs (Equation 3).


(9) $$SCC_{l}{}_{ncR-mR}=CC_{l}{}_{ncR-mR}-PCC_{l}{}_{ncR-mR}$$

Where PCC_lncR-mR_ is the partial canonical correlation (Equation 4) between the group of lncRNAs and mRNAs in the module, including the effect of shared miRNAs.


(10) $$PCC_{lncR-mR}=\frac{CC_{l}{}_{ncR-mR}-CC_{miR}{}_{-mR}CC_{miR}{}_{-lncR}}{\sqrt{1-CC_{miR-mR}^{2}}\sqrt{1-CC_{miR-lncR}^{2}}}$$

Modules with high canonical correlation were tested for significance with the null method model (List et al., 2019). The null model method hypothesizes that the miRNAs do not influence the canonical correlation between the group of lncRNAs and mRNAs (SCC_lncR-mR_ = 0). For the null model, the number of sampled datasets is 1E+6. A module with an adjusted *p*-value (adj *p*-value) < 0.05 (Benjamini-Hochberg correction method) is considered a miRNA sponge module.

The ME expression value of significant miRNA sponge modules was correlated with disorders (ASD, SCZ, and BD). We also validated the identified modules by performing module preservation analysis on the independent brain and blood-transcriptomic datasets of ASD, SCZ, and BD. The modules are shown to be reproducible (or preserved) based on the preservation of connectivity patterns of modules from reference networks in the different test networks (Langfelder et al., 2011). Density-based preservation statistics are used to find which module nodes remain highly connected in the test network. A connectivity-based preservation statistics are used to evaluate whether the connectivity pattern between nodes in the reference network is comparable to the test network. A preservation statistic is obtained by aggregating the density-based and connectivity-based preservation statistics. We accomplished this using the WGCNA package “module preservation” in R. The mean and variance of the preservation statistic were computed by random 200 permutations of module labels in the test network. Z statistic is defined by standardizing the preservation statistics with mean and variance. All individual Z statistics are aggregated by (Equation 5).


(11) $$Z_{summary}=\frac{Z_{density}+Z_{connectivity}}{2}$$

A *Z*_summary_ > 10 is considered strong evidence for module preservation, a value between 2 and 10 (2 < *Z*_summary_ < 10) is considered weak to moderate evidence of preservation and a *Z*_summary_ < 2 is considered no evidence of preservation (Langfelder et al., 2011). Additionally, we also examined whether the preserved modules show a strong canonical correlation between the lncRNAs and mRNAs in the test brain and blood-based transcriptome datasets.

### Functional enrichment analysis

The GO terms and KEGG pathways associated with each module were obtained using WebGestalt (Over-representation analysis) (Wang et al., 2017) with a background list of genes expressed in 50% of samples. To correct for multiple hypothesis testing, Benjamini–Hochberg method was used for GO terms and KEGG pathways. An adj *p*-value threshold of less than 0.05 was applied to find significant biological processes and pathways.

### ROC curve analysis

The lncRNAs of the identified modules can be a good candidate as diagnostic biomarkers. We obtained the expression level of the lncRNAs from miRNA sponge modules and used the pROC package in R to calculate the area under the ROC (receiver operating characteristic) curve and to plot the ROC curves.

## Results

### Pan-neuropsychiatric disorder miRNA sponge modules

The normalized pan-neuropsychiatric disorder gene expression data contains 14,030 mRNAs, 2,671 lncRNAs, and 35 miRNAs. We performed WGCNA on the expression data of mRNAs and lncRNAs. A gene co-expression network was constructed, and we identified 29 co-expressed modules. The miRNA sponge modules were identified based on three conditions: significant sharing of miRNAs, high canonical correlation, and sensitivity canonical correlation conditioning on shared miRNAs. We first selected 25 of these 29 modules based on the criteria that a miRNA sponge module should have at least three lncRNAs.

**Figure 2:**
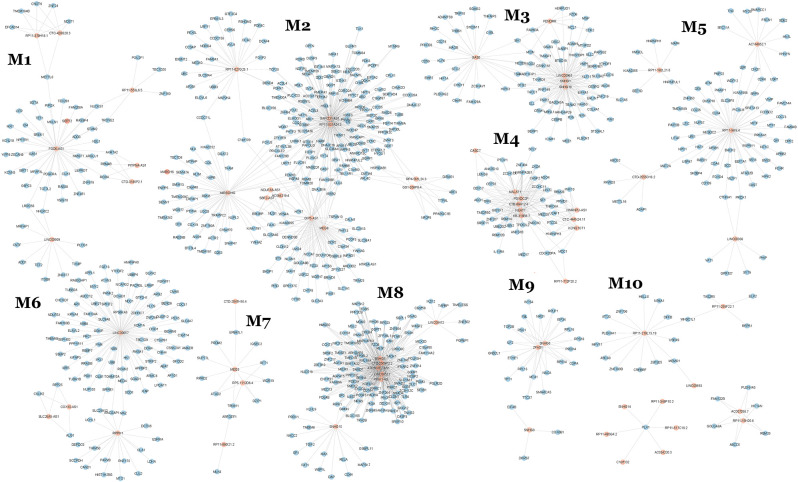
lncRNA-related miRNA sponge modules of pan-neuropsychiatric disorders.

**Figure 3:**
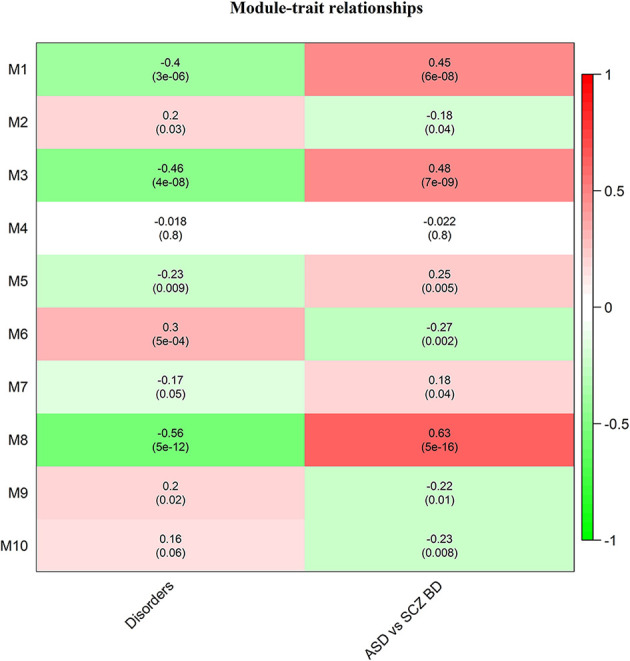
Heatmap of correlation between the disease traits and module eigengene. Correlation with disorders is calculated based on ASD-1, SCZ-2, and BD-3, and ASD vs. SCZBD is calculated based on ASD-1 and SCZBD-0. The *p*-values are given in the bracket.

Of these, 10 modules were identified as miRNA sponge modules with significant sharing of miRNAs and high canonical correlation (adj *p*-value < 0.05; Supplementary Table S3). This approach predicted 1,705 ceRNA interactions between 67 lncRNAs and 782 mRNAs from the 10 modules (Table 1). Figure 2 shows the ceRNA interactions within each significant module. These modules are associated with 10 overlapping miRNAs that target more than one module (Supplementary Data S1). hsa-miR-421, hsa-miR-1299, hsa-miR-3167, and hsa-miR-4705 are the most common miRNAs shared between modules. The association between the identified modules and the clinical traits was explored. The ME expression value of some of the modules shows a significant correlation with disease (*p*-value < 0.05; Figure 3). M1, M3, and M8 modules show a positive correlation with ASD. These modules are upregulated in ASD compared to SCZ and BD conditions (Supplementary Figure S1).

**Table 1:** miRNA sponge modules of pan-neuropsychiatric disorders and the number of miRNA-sponge interactions in each module.

**Modules**	**miRNAs**	**lncRNAs**	**mRNAs**	**Putative miRNA-lncRNA**	**Putative miRNA-mRNA**	**lncRNA-related miRNA sponge interactions**
M1	5	78	421	8	65	75
M2	7	213	1,624	13	279	470
M3	5	25	553	8	89	180
M4	8	299	331	23	48	178
M5	5	73	600	5	90	90
M6	5	107	1,189	6	109	111
M7	4	68	404	5	13	18
M8	3	70	766	7	121	505
M9	3	12	184	4	24	41
M10	5	532	361	12	25	37

The differentially expressed mRNAs and lncRNAs between the control and disease groups for ASD, SCZ, and BD conditions were retrieved from Gandal et al. (2018a) and mapped to the identified modules. This helps to identify modules containing disease-specific genes whose pattern of expression may vary across disorders. The differentially expressed mRNAs are majorly distributed across the modules M2, M3, M6, and M8 (Supplementary Table S4). Modules with the maximum number of differentially expressed lncRNAs are M2, M4, M6, and M10 (Supplementary Table S5). In the M2 module, 23 lncRNAs are differentially expressed in ASD conditions, including OIP5-AS1 and MIR600HG. OIP5-AS1 is downregulated in ASD patients, and its mRNA interactors are associated with the Hippo signaling pathway and glutamatergic synapse (Figure 2). MIR600HG is also downregulated in ASD patients, and its interactors are associated with the Wnt signaling pathway, TGF-beta signaling pathway, and longevity regulating pathway. On the other hand, SBF2-AS1 from the M2 module is a downregulated lncRNA in SCZ patients, and its interacting mRNAs are associated with serotonergic synapse and necroptosis. In the M1 module, the lncRNA FGD5-AS1 and LINC00909 are downregulated in SCZ patients. The mRNA interactors of FGD5-AS1 are associated with the JAK-STAT signaling pathway and circadian rhythm. The mRNA interactors of LINC00909 are associated with apoptosis and interferon-alpha response. LINC00511 of M8 is upregulated in SCZ, and its mRNA interactors are associated with proteoglycans in cancer, spliceosome, and fatty acid elongation.

Further, the lncRNAs with the highest out-degree in different modules were identified. This includes lncRNAs SAPCD1-AS1 (M2), SNHG16 (M3), RP11-9819.4 (M5), PINK1-AS (M8), and LINC00657 (M6) (Figure 2). The mRNA interactors of these lncRNAs map to axon guidance and neurotrophin signaling pathway (SAPCD1-AS1), pathways in cancer (PINK1-AS, SNHG16), p53 signaling pathway (RP11-9819.4, SNHG16) and neurodegeneration (LINC00657). LINC00893 from M10 is found to be interacting with MEMO1, which controls the radial unit development and neuronal laminar structure *via* regulating radial glial cell tiling. MEMO1 mutations and the resulting cortical abnormalities may increase the risk of autism (Nakagawa et al., 2019).

### Preservation of miRNA sponge modules

**Figure 4:**
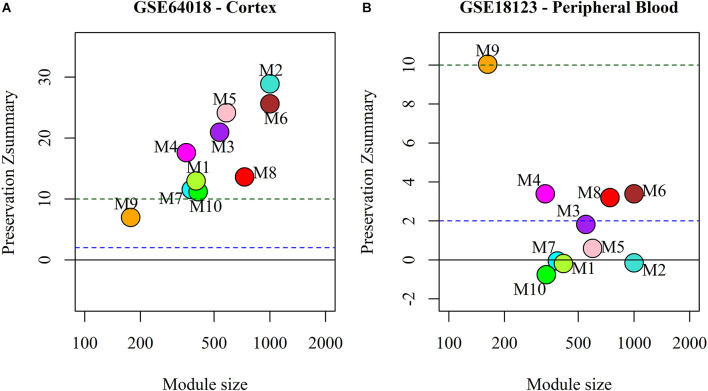
The preservation of miRNA sponge modules in **(A)** ASD cortex and **(B)** PBMC datasets.

**Figure 5:**
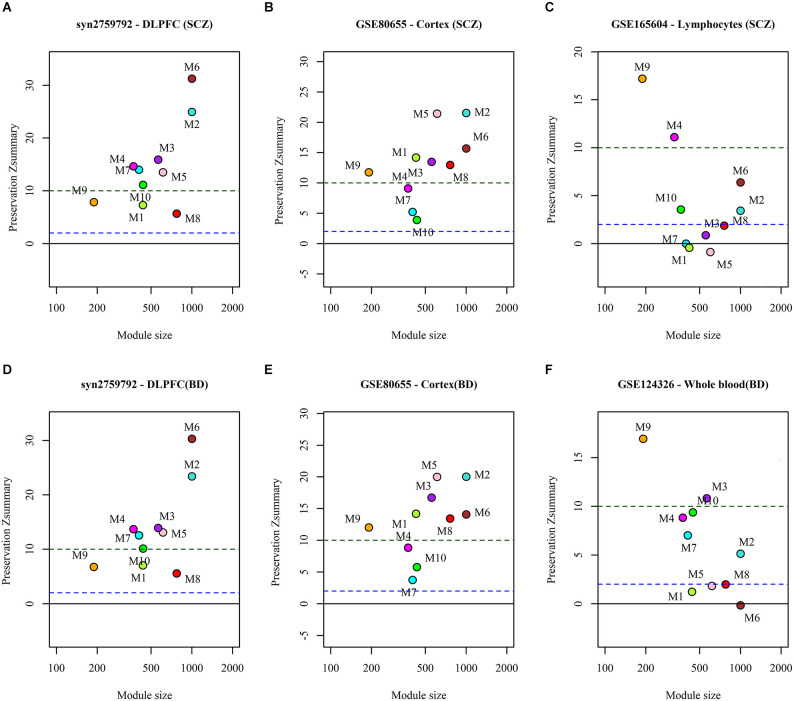
The preservation of miRNA sponge modules in the post-mortem brain and blood-based datasets of SCZ (top panel: **A,B,C**) and BD (bottom panel: **D,E,F**).

We analyzed the preservation of the identified miRNA modules in post-mortem brain and blood-based samples of ASD, SCZ, and BD patients (Table 2). The modules show moderate to high preservation in the test brain transcriptome data of ASD, SCZ, and BD patients with *Z*_(summary)_ > 2 (Figures 4 and 5). In the blood-based transcriptome of SCZ and BD, modules M2, M4, M9, and M10 show moderate to high preservation. Module M6 is preserved only in SCZ, and modules M3 and M7 are preserved only in BD blood-based transcriptome profiles. Modules M4, M6, M8, and M9 show moderate to high preservation in the PBMC dataset of ASD. The conserved miRNA sponge modules also show a significant canonical correlation between lncRNAs and mRNAs in post-mortem brain and blood-based transcriptomes. Modules M1 to M6, M9, and M10 are significant in ASD, while modules M1 to M4, M6, and M8 to M10 are significant in SCZ and/or BD (adj *p*-value < 0.05; Table 2).

**Table 2:** Modules showing moderate to high preservation and significant canonical correlation of lncRNAs and mRNAs in the test post-mortem brain and blood-based transcriptome.

**Datasets**	**Sample**	**Canonical correlated modules**
ASD—GSE64018	Cortex	M1–M6, M9, and M10
SCZ—GSE80655	Post-mortem brain region	M1 and M8
BD—GSE80655	Post-mortem brain region	M1 and M8
ASD—GSE18123	Peripheral blood	M4, M6, and M9
SCZ—GSE165604	Lymphocytes	M6 and M10
BD—GSE124326	Whole blood	M2, M3, M4, M9, and M10

### miRNA sponge modules associated pathways and biological processes

We identified the KEGG pathways and biological processes associated with the miRNA sponge modules using WebGestalt (Supplementary Data S1). The ASD upregulated module M1 is related to fatty acid oxidation and amino acid metabolism. Abnormal β-oxidation of unsaturated fatty acids has been reported in ASD (Clark-Taylor and Clark-Taylor, 2004). Module M2 is associated with chemical synaptic transmission, nervous system development, and KEGG pathways, Axon guidance and Wnt signaling pathway. The ASD upregulated modules M3 and M8 are associated with pathways in cancer, cytokine-mediated signaling pathways, and immune system processes. The pathways in cancer include genes involved in cell adhesion, oxidative stress, MAPK signaling, and apoptosis. Cell adhesion gene CTNNA2 in the module M8 encodes α-catenin, which plays a role in synaptic plasticity, and is associated with BD and SCZ (Terracciano et al., 2011). It is strongly expressed in the central nervous system, but its expression significantly changes in the post-mortem brain samples of SCZ. Increasing the expression or function of CTNNA2 is proposed as a potential therapeutic strategy for NPDs (Eszlari et al., 2021). NFE2L2 in the module M8 encodes Nrf2, which plays a role in cellular antioxidant response. NQO1, a target of Nrf2, is also a part of module 8. BD and SCZ are linked to persistent oxidative and nitrosative stress. Increasing Nrf2 activity is proposed as a therapeutic treatment for NPDs (Morris et al., 2021).

Modules M3 and M8 include different MAPKs and members of the Frizzled gene family. In module M3, MAPKs are co-expressed with GADD45 s, which are known to act through the MAPK cascade to control the response to stress signals. Increased activity of the MAPK signaling pathway is shown in ASD (Rosina et al., 2019). The knockdown of GADD45a reduces the effect of mood stabilizer Valproic acid (Yamauchi et al., 2007), and GADD45a regulates the expression of brain-derived neurotrophic factor (BDNF) (Feng et al., 2021). Module M3 also includes genes of HIF-1 signaling pathway genes, NFKβ signaling pathway, TNF signaling pathway, and cytokine-cytokine receptor interaction. The expression of immune-receptor genes (M3) is lower in SCZ, and BD compared to ASD (Supplementary Figure S2). Proinflammatory cytokines (TNF-α, IL-6) are shown to be significantly increased in the brain of ASD patients (Li et al., 2009).

Module M7 is associated with glutamatergic synapse, oxytocin signaling pathway, GABAergic synapse, and cholinergic synapse. Module M6 is associated with metabolic pathways and ubiquitin-mediated proteolysis. This module includes genes from glycolysis, the TCA cycle, and oxidative phosphorylation (Supplementary Data S1). We also observed that genes of module M6 map to pathways of neurodegeneration. Module M9 is associated with the ribosome, protein export, protein processing in ER, and ncRNA processing.

### lncRNAs as potential biomarkers for neuropsychiatric disorders

**Figure 6:**
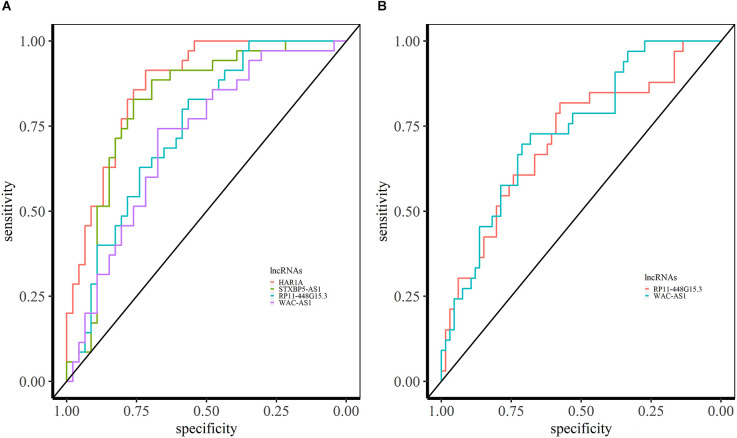
ROC analysis based on lncRNA expression in ASD. **(A)** ROC curves of HAR1A, STXBP5-AS1, RP11-448G15.3, and WAC-AS1 in syn11242290 (ASD—pancortical). **(B)** ROC curved of RP11-448G15.3 and WAC-AS1 with AUC > 0.70 in PBMC.

We performed a ROC curve analysis to find the lncRNAs which can be the potential diagnostic biomarker. We obtained the expression levels of lncRNAs from the identified miRNA sponge modules. In the ASD- pan-cortical data, we identified 126 lncRNAs with the area under the ROC (AUC) > 0.7 (Supplementary Data S1). Of these, 40 lncRNAs have AUC value > 0.7 in both ASD pan-cortical and GSE64018 (ASD—cortex). HAR1A from M6 is a candidate lncRNA for ASD with the best AUC value of 0.88 (Figure 6A). HAR1A is associated with the central nervous system, Huntington’s disease, Alzheimer’s disease, and SCZ. It is also associated with ASD, impulsive behavior, and ADHD (Piñero et al., 2017; Rappaport et al., 2017). STXBP5-AS1 (M2) has an AUC of 0.83 in ASD brain datasets and is also differentially expressed in PBMC of ASD patients (Wang et al., 2015; Tang et al., 2017). RP11-448G15.3 (M8) and WAC-AS1 (M2) have an AUC value > 0.70 in both brain and blood-based datasets and can be the diagnostic biomarkers for ASD (Figure 6).

**Figure 7:**
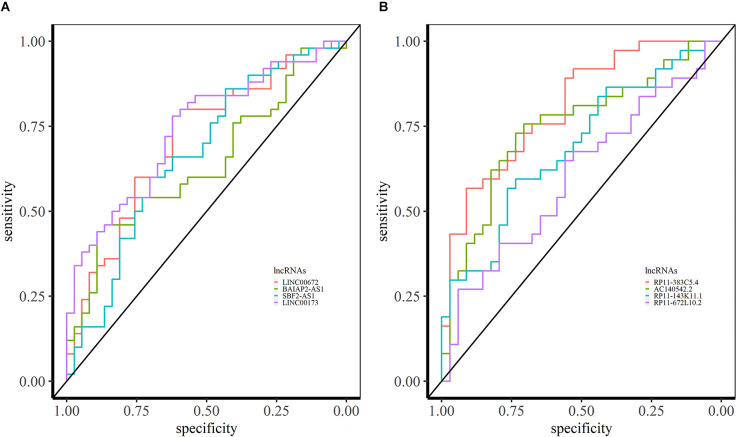
ROC analysis based on lncRNA expression in SCZ and BD. **(A)** ROC curves of LINC00672, BAIAP2-AS1, SBF2-AS1, and LINC00173 in BrainGVEX SCZ patients. **(B)** ROC curves of lncRNAs with AUC > 0.6 in BrainGVEX BD patients.

In SCZ, we identified 64 overlapping lncRNAs across different brain datasets (BrainGVEX, CMC, and GSE80655) with AUC > 0.6 (Supplementary Data S1). The candidate LINC00672 of M2 has an AUC value of 0.69 (in BrainGVEX) and is also associated with Alzheimer’s disease (Rappaport et al., 2017) (Figure 7A). BAIAP2-AS1 (M2) module has an AUC value of 0.60 in BrainGVEX and 0.72 in lymphocytes data of SCZ patients. McKinney et al. (2017) reported the hypomethylation of BAIAP2-AS1 in SCZ patients, which may result in increased transcription of BAIAP2-AS1. SBF2-AS1, a downregulated lncRNA in SCZ from M2, has an AUC value of 0.67 in BrainGVEX and 0.84 in lymphocytes. LINC00173 (M10) has an AUC value of 0.74 in BrainGVEX and 0.65 in lymphocytes.

Eight overlapping lncRNAs were identified with an AUC value greater than 0.6 across different brain-based transcriptomic datasets of BD. The best candidate lncRNA RP11-383C5.4 from M6 has an AUC value of 0.81 in BrainGVEX data (Figure 7B). Twenty-three lncRNAs have AUC values greater than 0.6 in both BrainGVEX and whole blood transcriptome. The blood-based candidate lncRNAs with high AUC values include AC140542.2 (M6), RP11-143K11.1 (M2), RP11-672L10.2 (M2), and ZFAS1 (M9).

## Discussion

We investigated the role of lncRNAs as miRNA sponges in NPDs (ASD, SCZ, and BD). A pan-neuropsychiatric disorder transcriptome of ASD, SCZ, and BD patients was analyzed to construct the gene co-expression network. Using a canonical correlation-based approach, we identified 10 modules as significant miRNA sponge modules. We identified the biological processes and pathways associated with the modules. The preservation of these modules in other post-mortem brain and blood-based transcriptomes was studied. We also investigated the diagnostic potential of the lncRNAs from these modules by performing ROC analysis using their expression level in both the post-mortem brain and blood-based transcriptome studies. The identified modules showed similar and distinct gene expression patterns depending on the neuropsychiatric condition (Figure 3). Our approach identified 1,477 lncRNAs as miRNA sponging RNAs (Table 2) based on co-expression patterns, providing information on the associated biological processes.

The miRNA sponge modules of NPD are associated with metabolic, inflammatory, and cancer pathways, which show a distinct pattern of expression. These are upregulated in ASD compared to SCZ and BD (Supplementary Figure S1). Module M1 is associated with metabolic pathways (fatty acid oxidation, amino acid metabolism). Recent evidence shows that ASD, SCZ, and BD are associated with metabolic abnormalities (Clark-Taylor and Clark-Taylor, 2004; Gillberg et al., 2017; Moolamalla and Vinod, 2020). The lncRNA FGD5-AS1 from the M1 module (Figure 2) is shown to act as a competing endogenous RNA protecting against neuron injury (Zhang X. Q. et al., 2019). The genes from modules M3 and M8 are related to inflammatory pathways and show a difference between NPDs (Supplementary Figure S1). This is consistent with the observation that immune dysfunction plays a role in neurodevelopmental deficits in autism (Hughes et al., 2022) and SCZ (Murphy et al., 2021). Gandal et al. (2018a) showed that the microglial cell enriched module is upregulated in ASD and downregulated in SCZ and BD. As opposed to the episodic nature of active psychosis in SCZ, the clinical pattern of ASD patients is marked by relative symptom continuity. In studies of post-mortem brain and peripheral blood samples of ASD, immunological abnormalities have been found in individuals of all ages, pointing to a persistent immune activation that worsens with the severity of symptoms (Michel et al., 2012).

Interestingly, inflammatory pathways are co-expressed with pathways in cancer in modules M3 and M8. The risk genes and pathways for ASD and cancer are reported to be similar (Forés-Martos et al., 2019). Module M3 includes lncRNA SNHG1 (Figure 2), which has been previously linked to neuroinflammation in Parkinson’s disease (PD) and cell proliferation in cancer *via* miRNA sponging (Tian et al., 2017; Cao et al., 2018). The lncRNA GAS5 from module M3 suppresses inflammatory responses and apoptosis of alveolar epithelial cell MLE-12 by targeting the miR-429/DUSP1 axis. GAS5 sponges the miRNA miR-429 and facilitates the DUSP1 expression (Li and Liu, 2020). DUSP1 (M3) is co-expressed with GAS5 in the pan-NPD transcriptome. However, miR-429 is not expressed in 50% of samples and is eliminated during pre-processing. DUSP1 is involved in the regulation of anti-inflammatory genes, and it is associated with mental disorders including BD, major depressive disorder, Alzheimer’s disease, Huntington’s disease, and cognition disorder (Piñero et al., 2017). PINK1-AS from module M8 is linked to PD and has a neuroprotective role against stress-induced mitochondrial dysfunction (Policarpo et al., 2021). hsa-miR-421 and hsa-miR-3167 are two miRNAs shared by M1, M3, and M8 modules. hsa-miR-421 is shown to be upregulated in BD (Pisanu et al., 2019).

Although modules M2, M6, M7, and M9 did not show a higher correlation with disorders compared to M1, M3, and M8 modules, the pathway enrichment of modules captured relevant pathways associated with NPDs. We observed that the eigengene expression of these modules shows heterogeneous patterns within disease groups. The modules M2 and M7 are linked to neuron-specific pathways connected to synapses. Module M2 is associated with the neuronal development pathway, Wnt signaling, which controls many key functions throughout the development of the vertebrate central nervous system, including patterning and cell fate specification, proliferation, and neuronal morphology. The Wnt signaling pathway regulates neurite outgrowth, axon remodeling, synapse formation and plasticity, and neurogenesis in the adult brain (Valvezan and Klein, 2012). Hoseth et al. (2018) found that the Wnt signaling pathway is disrupted in SCZ and BD patients, and they hypothesized that medications targeting the Wnt pathway could help cure mental illnesses (Hoseth et al., 2018). The WNT/β-catenin pathway is also dysregulated in ASD (Vallée and Vallée, 2018). The lncRNA LINC00617/ TUNA from module M2 can affect the gene expression in neuronal cells, and the knockout of TUNA is shown to inhibit neuronal differentiation in mouse embryonic stem cells (Lin et al., 2014). Chen et al. (2021) reported the sponging effect of OIP5-AS1 (from the module M2) with miR-186-5p, and this activity protects neuron injury against cerebral hypoxia–ischemia-induced inflammation and oxidative stress.

Module M9 is linked to the ribosome and protein export. Studies show that the lncRNAs SNHG6 and ZFAS1 from M9 (Figure 2) are the ribosome-linked lncRNAs (Hansji et al., 2016; Birgani et al., 2018). The lncRNA landscape obtained from brain tissues of SCZ patients shows that the ribosome and protein synthesis pathway is upregulated. The ribosome protein genes are also upregulated in ASD post-mortem cortical tissues and induced pluripotent stem cell (iPSC)-derived neural progenitor cells of ASD (Tian et al., 2018; Lombardo, 2020). The lncRNA SNHG6 functions as a ceRNA to regulate neuronal apoptosis in ischaemic stroke (Zhang X. et al., 2019). The lncRNA MIAT from module M7 is a well-known lncRNA linked to NPD (Rusconi et al., 2020). The MIAT level decreases with neuronal activation, and it facilitates a pro-anxiety transcriptional program. The lncRNA MEG3 from module 7 functions as a ceRNA in ischemia-induced neuronal cell apoptosis (Liu et al., 2016). MEG3 is upregulated, and PINT (from module M10) is downregulated in SCZ post-mortem brain samples (Ghafouri-Fard et al., 2021).

The role of lncRNAs OIP5-AS1 (M2), SBF2-AS1 (M2), MIAT (M7), MEG3 (M7), SNHG5 (M8), LINC00511 (M8), SNHG6 (M9) as miRNA sponges are well-studied in cancer (Wang D. et al., 2020, Wang H. S. et al., 2020; Lu et al., 2021; Zhang S. et al., 2021). Other well-known lncRNAs NEAT1 (M4) and MALAT1 (M4) in NPD are present in module M4, which does not show a significant correlation with the disease but shows higher module preservation in blood-based transcriptomic data (Rusconi et al., 2020). We predicted the potential biomarkers for NPD based on the expression level of lncRNA in brain tissue and blood. The lncRNA-based approach yielded higher AUCs for ASD than BD and SCZ (Figures 6 and 7). The analysis also revealed the preservation of co-expression patterns across brain and blood samples in NPD, suggesting common pathological mechanisms.

Overall, our study provides a detailed landscape of lncRNAs in NPD and their role as competitive endogenous RNAs using a modular approach. The prior studies on the ceRNA network in NPD mapped differentially expressed mRNAs and lncRNAs to the putative interactions from the databases (He et al., 2021; Li et al., 2021; Sabaie et al., 2021a, b). However, these studies do not consider the co-expression pattern of miRNA, lncRNA, and mRNA, and all the potential RNAs may not be differentially expressed. We performed a large-scale inference of the lncRNA-related miRNA sponge network in the NPD, which captured the co-expressed miRNA sponge RNAs (lncRNAs and mRNAs) across the disorders. The miRNA sponge activity of lncRNAs shows cross-disorder expression overlap and conservation in the post-mortem brain and blood-based samples. We provide the curated list, including interactions involving lncRNAs as miRNA sponges and their associated biological processes and potential biomarkers for NPDs (Supplementary Data S1). It will serve as a valuable resource for further exploration by experiments.

## Data Availability Statement

The datasets presented in this study can be found in online repositories. The names of the repository/repositories and accession number(s) can be found in the article/Supplementary material.

## Ethics Statement

Ethical review and approval was not required for the study on human participants in accordance with the local legislation and institutional requirements. Written informed consent for participation was not required for this study in accordance with the national legislation and the institutional requirements.

## Author Contributions

PV: conceptualization, funding acquisition, and supervision. RB: methodology, formal analysis, investigation, and writing—original draft preparation. RB and PV: writing—review and editing. All authors contributed to the article and approved the submitted version.

## Funding

This work was supported by iHUB-Data, International Institute of Information Technology, Hyderabad, India.
