# Supplementary material for: Inferring miRNA sponge modules across major neuropsychiatric disorders
Source: Front Mol Neurosci. 2022 Oct 28;15:1009662. doi: 10.3389/fnmol.2022.1009662 (PMC9650411; doi:10.3389/fnmol.2022.1009662)
Supplement: Supplementary file 2 [file Data_Sheet_2.PDF]

Supplementary Information

INFERRING miRNA SPONGE MODULES ACROSS MAJOR NEUROPSYCHIATRIC DISORDERS

Rami Balasubramanian and P K Vinod*

Centre for Computational Natural Sciences and Bioinformatics, International Institute of Information Technology, Hyderabad-500032, India

*Correspondence: vinod.pk@iiit.ac.in

Supplementary figures

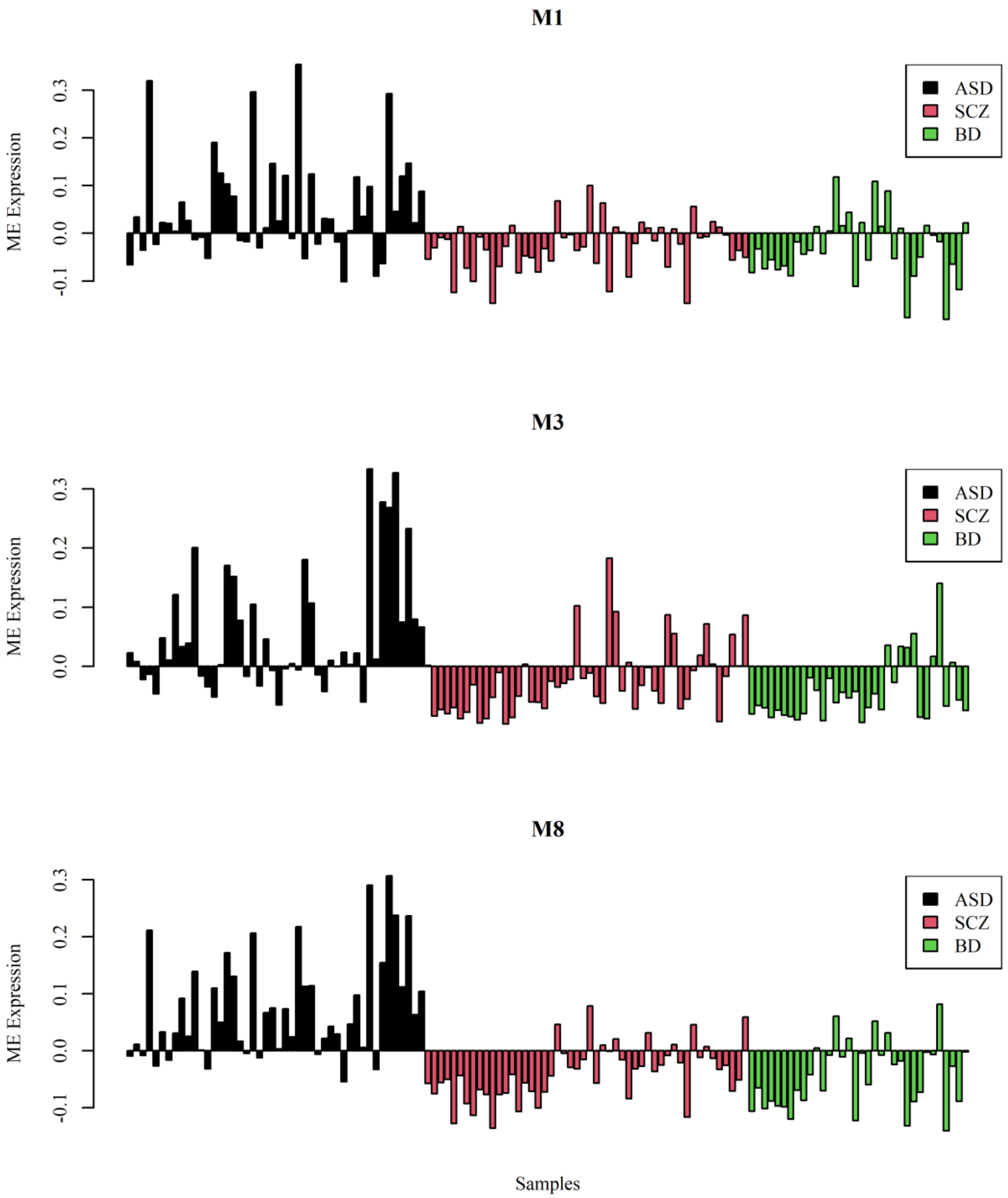

Figure S1: Eigen gene expression of Module M1, M3 and M8 across neuropsychiatric disorders (ASD, SCZ, and BD). These modules are upregulated in ASD and downregulated in SCZ and BD.

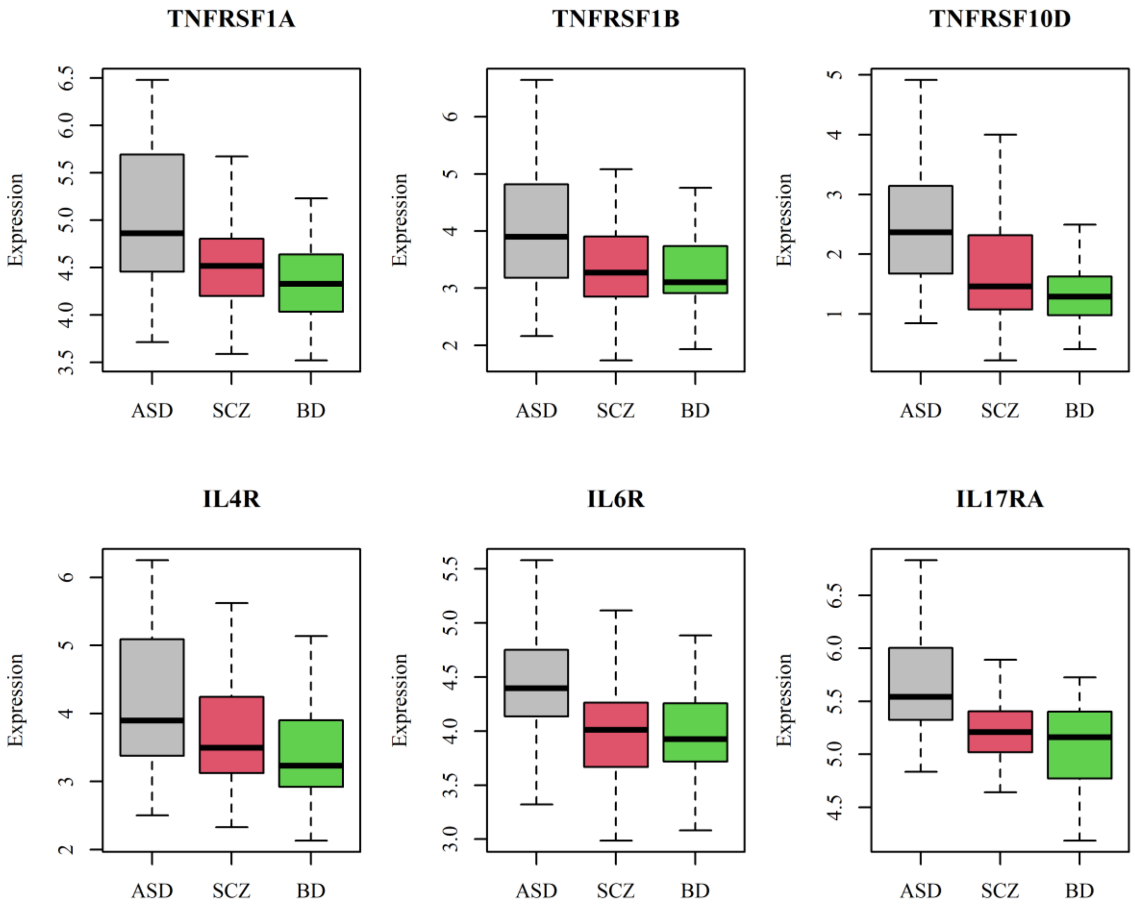

Figure S2: Expression of immune-receptor genes (module M3) across the ASD, SCZ, and BD samples.

Supplementary Tables

Table S1: Datasets used to build the pan-neuropsychiatric disorder transcriptome

Disease	Dataset	Samples	Synapse
ASD	ASD- pancortical	53	syn11242290
SCZ	BrainGVEX	53	syn4590909
BD	BrainGVEX	47	syn4590909

Table S2: Post-mortem brain and blood-based transcriptome data used to perform module preservation and ROC analysis

**Dataset**	**Disease**	**Sample source**	**Sequencing**	**Samples**
GSE80655	SCZ, BD, MDD	Anterior cingulate gyrus, dorsolateral prefrontal cortex, and nucleus accumbens	RNA-seq	281 post-mortem brain tissues from 24 patients in each condition
GSE64018	ASD	Cortex	RNA-seq	12 -ASD<br>12- Control
syn2759792	SCZ and BD	DLPFC	RNA-seq	262 SCZ, 47 BD and 295 CTL
GSE165604	SCZ	Lymphocytes	RNA-Seq	19 SCZ and 18 CTL
GSE124326	BD with and without lithium treatment	Whole blood	RNA-Seq	240 BD and 240 CTL
GSE18123	ASD	Peripheral blood	Affy HG-U133 plus2	66 ASD and 33 CTL

Table S3: The canonical correlation and sensitivity canonical correlation of the identified miRNA sponge modules.

Module	cor	pcor	mscor	p.val	p.adj
M1	0.937075	0.901968	0.035108	0.000847	0.002372
M2	0.97459	0.935466	0.039124	0.000331	0.001159
M3	0.926062	0.878979	0.047084	4.40E-05	0.000308
M4	0.982307	0.966062	0.016245	0.031188	0.043663
M5	0.953445	0.931607	0.021838	0.011928	0.018841
M6	0.964795	0.936347	0.028447	0.003342	0.007798
M7	0.958022	0.93627	0.021752	0.012112	0.018841
M8	0.886484	0.788887	0.097597	1.00E-06	1.40E-05
M9	0.891262	0.867286	0.023976	0.007947	0.015894
M10	0.981764	0.941848	0.039916	0.000284	0.001159

Table S4: Differentially expressed mRNAs across the modules.

Module	ASD DEGs	SCZ DEGs	BD DEGs
M1	26	39	6
M2	296	178	18
M3	181	113	4
M4	8	33	7
M5	13	35	5
M6	243	140	19
M7	25	21	4
M8	171	81	14
M9	5	4	0
M10	13	20	4

Table S5: Differentially expressed lncRNAs across the modules.

Module	ASD DEGs	SCZ DEGs	BD DEGs
M1	0	1	0
M2	23	16	4
M3	0	1	0
M4	6	20	1
M5	0	0	0
M6	9	6	1
M7	3	0	0
M8	5	1	0
M9	0	0	0
M10	10	12	6

Data S1: List of lncRNAs, mRNAs and lncRNA-related miRNA sponge interactions, and their association to functional pathways in neuropsychiatric disorders (excel sheet). The lncRNA’s predictive AUC values are also included.
